# Factors associated with research productivity in higher education institutions in Africa: a systematic review

**DOI:** 10.12688/aasopenres.13211.1

**Published:** 2021-05-26

**Authors:** Dieudonne Uwizeye, Florah Karimi, Caroline Thiong'o, Jackline Syonguvi, Vollan Ochieng, Francis Kiroro, Alex Gateri, Anne M. Khisa, Hesborn Wao

**Affiliations:** 1 Department of Development Studies, University of Rwanda, Kigali, Rwanda; 2 African Population and Health Research Center, Nairobi, Kenya

**Keywords:** Research productivity, factors associated to research, institutional factors, motivations, higher education institutions, Africa

## Abstract

**Background**: There are low levels of research productivity among Higher Education Institutions (HEIs) in Africa, a situation that is likely to compromise the development agenda of the continent if not addressed. We conducted a systematic literature review to determine the factors associated with research productivity in HEIs in Africa and the researchers’ motives for research.

**Methods**: We identified 838 papers related to research productivity in HEIs in Africa from various databases, from which we included 28 publications for review. The inclusion criteria were that (i) the paper’s primary focus was on factors associated with research productivity; (ii) the setting was on the higher education institutions in Africa; (iii) the type of publication was peer-reviewed papers and book chapters based on primary or secondary data analysis; and (iv) the language was English or French. Essays, opinions, blogs, editorials, reviews, and commentaries were excluded.

**Results**: Most of the studies operationalized research productivity as either journal publications or conference proceedings. Both institutional and individual factors are associated with the level of research productivity in HEIs in Africa. Institutional factors include the availability of research funding, level of institutional networking, and the degree of research collaborations, while individual factors include personal motivation, academic qualifications, and research self-efficacy.

**Conclusions**: Deliberate efforts in HEIs in Africa that addressed both individual and institutional barriers to research productivity are promising. This study recommends that the leadership of HEIs in Africa prioritizes the funding of research to enable researchers to contribute to the development agenda of the continent. Moreover, HEIs should build institutional support to research through the provision of research enabling environments, policies and incentives; strengthening of researchers’ capabilities through relevant training courses, mentorship and coaching; and embracing networking and collaboration opportunities.

## Introduction

There is a close association between research and development, both of which play a crucial role in economic growth ( Bayarçelik & Taşel, 2012; Blanco *et al.*, 2016). The United Nations, through the Sustainable Development Goals (SDGs), specifically, Target 9.5, have prioritized the enhancement of scientific research, particularly in developing countries ( Maiyo, 2015). Higher Education Institutions (HEIs) are well-suited to spearhead the realization of the global development agenda through research and innovations and the provision of expertise to guide the process ( El-Jardali *et al.*, 2018; The World Bank, 2007). Generally, HEIs contribute to generating innovative ideas to feed the development process ( Clegg, 2012). However, in most of the African countries, university faculty members are assessed mainly based on the modules/course they teach and the number of students they supervise than the research productivity ( Kpolovie & Dorgu, 2019).

Similarly, funding for research has remained low in most of the countries in Africa ( Saric *et al.*, 2018). A global assessment of the research and development expenditure, as a proportion of the Gross Development Product, reveals that many of the African countries invest less than 1% on research and development, the African Union target ( Karimi, 2015; Maiyo, 2015; UNESCO Institute of Statistics, 2018). Also, the number of African researchers was not proportional to the African population. Apart from Morocco, all the other African countries have less than 1000 researchers per one million inhabitants ( UNESCO Institute of Statistics, 2018).

The situation described above suggests that African countries need to re-consider their research agenda taking cognizance of the crucial role played by research in the development agenda ( Mwendera *et al.*, 2017), and the contribution of the HEIs to research and knowledge creation ( Clegg, 2012). Therefore, there is an urgent need to determine the factors that contribute to research productivity in HEIs in Africa to inform the directions of improving the research landscape within the African region. The purpose of this study is twofold: 1) to determine the factors associated with research productivity in higher education institutions in Africa; and, 2) to identify what motivates researchers working in HEIs in Africa to do research. This article is reported in line with the Preferred Reporting Items for Systematic Reviews and Meta-Analyses (PRISMA) ( Uwizeye *et al.*, 2021).

## Methods

We conducted a systematic review of publications from 1998 to 2018. The 20 years was selected to capture the changes that happened over the years as well as provide an opportunity to cover current knowledge to inform the development of research in HEIs in Africa.

### Inclusion and exclusion criteria

The selection of papers considered four criteria:

1. *Scope of the research:* Papers with a primary focus on factors associated with research productivity.

2. *The setting*: Higher education institutions in Africa.

3. *Type of publications*: Papers, books, and book chapters produced through the review process, based on primary or secondary data analysis. Essays, opinions, blogs, editorials, reviews, and commentaries were excluded.

4. *Language:* We targeted publications in English or French.

### Searching and selection of the studies

The search for publications involved two approaches:

***1.* Systematic search through EBSCO host**: We selected the leading databases in education hosted in EBSCO Host, namely Education Resources Information Center (ERIC), Education Search Complete, and Academic Search Ultimate, and we activated the advanced search. The search string was the following: Research product* OR research output OR publication* AND Higher education institution* OR tertiary institution* AND Africa*. Search limiters were *Scholarly (Peer Reviewed) Journals*, and the Publication dates were *January 1998 to December 2018*. Source Types were *Academic Journals* and the subject was limited to *higher education*. The systematic search was conducted in the last week of March, 2019.

**2. Search in other sources:** We conducted an additional search in the databases of the journals that occasionally publish education content, namely: Social science citation index, British education index, Web of Science, Scopus, Google Scholar, African Journals Online (AJOL), DOAJ, and EMERALD. The search string was the following: *"Factors" associated with "research productivity" in higher education institutions in "Africa".* The search in the other sources was done in April, 2019. Examples of the search outputs can be found as extended data ( Uwizeye *et al.*, 2021).

We worked in pairs at every stage of the selection process. Any disagreements on whether a study is to be included or excluded, a third member of the review team would read the paper and work with the team to reach a consensus.

### Data extraction

We developed a data extraction form to collect data on five primary indicators:

a) *Identification of the paper*: The study citation, location of the study, participant characteristics, and the source of funding.

b) *Methodology*: Design of the study, including the type of the study, methods of sampling, and sample size.

c) *Concepts*: The way the studies had operationalized the concept of research productivity and the definition of research output.

d) *Factors associated with research productivity*: The tool considered factors that were significantly associated with research productivity (for quantitative studies) or the factors that were found to be most frequently or intensely indicated (for qualitative studies).

e) *Motives for generating research products*: this aspect aimed to establish the individual researchers’ motivations in conducting research.

### Analysis approach

To identify factors associated with research productivity, we first examined a pool of variables identified in the previous studies and grouped them according to their similarities for classification ( Box 1). We reviewed the groups, referring to various studies that investigated similar topics, including Bryman (2007); Kpolovie & Onoshagbegbe (2017); Mantikayan & Abdulgani (2018) and Musiige & Maassen (2015), to gain consensus on the category titles and the factors that fall in the various groups. The factors were broadly grouped as either individual-related or institutional-related, as presented in Box 1.

**Box 1. Factors associated with research productivity**

**Table:** 

*Individual related*	*Institutional related*
**Demographic characteristics:** *Gender* *Age* *Tenure status* *Academic discipline*	***Capacity support and partnerships:*** Membership in professional body Networking/ research collaboration Research mentorship/ coaching leadership structures Research time Friendly research environment/ leadership Supervision of postgraduate
**Researcher’s psychological factors:** *Attitude/perception of research* *Culture of research* *Job satisfaction* *Motivation* *Research self-efficacy*	***Research funding:*** Financial incentives to encourage research Research grants Consultancies
**Individual competencies:** *Experience as a researcher* *Qualification and research training* *Research style*	***Infrastructural research enabling support:*** Institutional administrative structure Administrative workload Policies including intellectual property policy Internet connectivity Office space Institutional Ownership Salary

The study used the Critical Appraisal Skills Programme (CASP) tools to assess the methodological quality of the included studies, to describe their quality rather than a basis for inclusion. The CASP screening questions we used are presented in Box 2. The results of the assessment were presented using the Cochrane Review Manager tool (RevMan 5.3), a tool that allows for generating a graphical presentation. CASP offers tools to critically assess the quality, validity and reliability of the published research, to enable researchers to decide whether the evidence in the published work are relevant ( Galdas *et al.*, 2015).

The included papers were quantitative and qualitative. The CASP tool for quantitative and qualitative studies consists of twelve (12) and ten (10) items, respectively, and uses a 3-point response scale: 'Yes,' 'Cannot tell' or 'No.'

**Box 2: CASP screening questions used to assess the methodological quality of the included studies**

**Table:** 

**Critical Appraisal Skills Programme (CASP) screening questions to assess quantitative studies**
1) **Question**: Did the study address a clear and focused question/issue? 2) **Design:** Is the research method (study design) appropriate for answering the research question? 3) **Selection:** Is the method of selection of the subjects clearly described? 4) **Bias:** Could the way the sample was obtained, introduce (selection) bias? 5) **Representative:** Was the sample of subjects, representative of the population? 6) **Power:** Was the sample size based on pre-study considerations of statistical power? 7) **Response rate**: Was a satisfactory response rate achieved? 8) **Valid and reliable**: Are the measurements (questionnaires) likely to be valid and reliable? 9) **Statistical significance:** Was the statistical significance assessed? 10) **Confidence interval:** Are confidence intervals given for the main results? 11) **Confounders**: Could there be confounding factors that the study has not considered? 12) **Application**: Can the results be applied to your organization?
**CASP screening questions to assess qualitative studies**
1) **Aim**: Was there a clear statement of the purpose of the research? 2) **Methodology**: Is a qualitative method appropriate? 3) **Design**: Was the research design appropriate to address the aims of the research? 4) **Recruitment**: Was the recruitment strategy appropriate to the aims of the research? 5) **Data**: Was the data collected in a way that addressed the research issue? 6) **Relationship**: Has the relationship between the researcher and participants been adequately considered? 7) **Ethics**: Have ethical issues been taken into consideration? 8) **Rigorous**: Was the data analysis sufficiently rigorous? 9) **Findings**: Is there a clear statement of the results? 10) **Valuable**: Does the study contribute to valuable existing knowledge in research?

## Results

**Figure 1.**
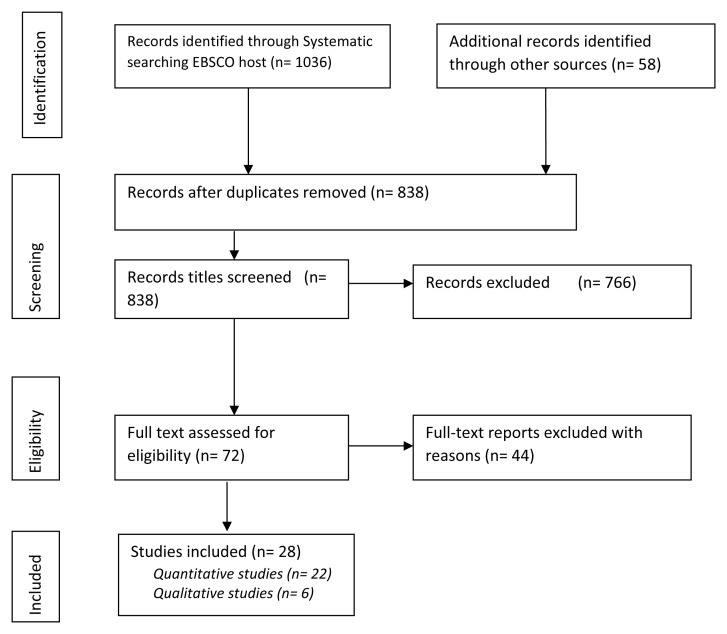
PRISMA flow diagram of literature search and selection process.

The search produced 1094 papers including 1036 identified through the systematic search in EBSCO host databases, and 58 new titles added from the search of other sources. We removed duplicates and remained with 838. Titles and abstracts were screened to ensure alignment to the inclusion criteria, and 766 abstracts were eliminated from the study, thus leaving 72 eligible papers for further scrutiny. We downloaded the 72 papers and read their entire texts to assess eligibility in line with the inclusion and exclusion criteria. We eliminated 44 of the publications mainly because the studies were not consistent with our inclusion criteria. We remained with 28 publications which were eventually scientific journal articles. Among these, 22 were quantitative studies, and 6 qualitative or mixed methods with a dominant qualitative approach. Figure 1 indicates the process of searching and identifying the papers.

Table 1 indicates that the selected studies reported from six African countries, with the highest number of papers conducted in South Africa and Nigeria (9, 32%, in each of the two countries), followed by Kenya with seven articles (25%). Other countries were Ethiopia, Uganda, and the United Republic of Tanzania, each with one study. Half (14, 50%) of the articles were conducted in single institutions, while thirteen (13, 46%) were from more than one HEI in a given country. In one study, it was not specified whether the data were collected from one or multiple HEIs.

**Table 1.** Study characteristics.

ID	Author, date	Location	Country	Funding source	Population	N	Age	Study design/ type	Type of data	Method of data collection	Intervention	Sampling Technique
**1**	Chepkorir, 2018	Single HEI	Kenya	Unspecified	Faculty	193	41	Quantitative	Primary	Survey	No	Simple Random
**2**	Okendo, 2018	Single HEI	Tanzania	Unspecified	Faculty	40	Not indicated	Mixed methods	Primary	Survey	No	Stratified
**3**	Snowball & Shackleton, 2018	Single HEI	South Africa (SA)	HEI	Faculty	174	Not indicated	Quantitative	Primary	FDG	No	Convenience
**4**	Weng’ua *et al*., 2017	Multi-HEI national	Kenya	Unspecified	Faculty	80	Not indicated	Mixed methods	Primary	Survey	No	Purposive
**5**	Callaghan, 2017	Single HEI	South Africa	Unspecified	Faculty	225	Not indicated	Quantitative	Primary	Survey	No	Purposive
**6**	Feyera *et al*., 2017	Single HEI	Ethiopia	HEI	Faculty	120	Not indicated	Quantitative	Primary	Survey	No	Stratified
**7**	Opesade *et al*., 2017	Single HEI	Nigeria	Unspecified	Faculty	115	Not indicated	Quantitative	Secondary	Desk review	No	Stratified
**8**	Moseti & Mutula, 2016	Multi-HEI national	Kenya	Unspecified	Faculty	605	Not Indicated	Quantitative	Primary	Survey	No	Simple Random
**9**	Muia & Oringo, 2016	Single HEI	Kenya	Unspecified	Faculty	58	41.3	Quantitative	Both	Survey	No	Purposive
**10**	Schafer *et al*., 2016	Multi-HEI national	Kenya	Unspecified	Faculty	473	Not indicated	Quantitative	Primary	Survey	No	Purposive
**11**	Okonedo, *et al*., 2015	Multi-HEI national	Nigeria	Unspecified	Librarians	142	41.82	Quantitative	Both	Survey	No	Purposive
**12**	Okonedo, 2015	Multi-HEI national	Nigeria	Unspecified	Librarians	142	Not indicated	Quantitative	Primary	Survey	No	Purposive
**13**	Ani *et al*., 2015	Multi-HEI national	Nigeria	Unspecified	Faculty	586	Not indicated	Quantitative	Primary	Survey	No	Stratified
**14**	Callaghan, 2015a	Single HEI	SA	Unspecified	Faculty	225	Not indicated	Quantitative	Primary	Survey	No	Purposive
**15**	Musiige & Maassen, 2015	Single HEI	Uganda	Unspecified	Faculty	9	Not indicated	Qualitative	Primary	Interview	No	Convenience
**16**	Anyaogu & Iyabo, 2014	Multi-HEI national	Nigeria	Unspecified	Faculty	414	42.4	Quantitative	Primary	Survey	No	Cluster
**17**	Nyaribo, 2014	Multi-HEI national	Kenya	Unspecified	Faculty	54	not indicated	Quantitative	Primary	Survey	No	Convenience
**18**	Okiki, 2013a	Multi-HEI national	Nigeria	Unspecified	Faculty	1057	Not indicated	Quantitative	Primary	Survey	No	Quota
**19**	Okiki, 2013b	Multi-HEI national	Nigeria	Unspecified	Faculty	873	Not indicated	Quantitative	Primary	Survey	No	Stratified
**20**	Obembe, 2012	Multi-HEI national	Nigeria	Unspecified	Faculty	77	46.12	Quantitative	Primary	Survey	No	Convenience
**21**	Sulo *et al*., 2012	Single HEI	Kenya	Unspecified	Faculty	242	Not indicated	Quantitative	Primary	Survey	No	Stratified
**22**	North *et al*., 2011	Single HEI	SA	HEI	Faculty	1178	43.4	Quantitative	Secondary	Survey	No	Database
**23**	Geber, 2009b	Single HEI	SA	Unspecified	Students	8	Not indicated	Qualitative	Primary	Interview	Yes	Purposive
**24**	Geber, 2009a	Single HEI	SA	Unspecified	Faculty	8	Not indicated	Qualitative	Primary	Interview	Yes	Purposive
**25**	Ogbogu, 2009	Multi-HEI national	Nigeria	Unspecified	Faculty	381	Not indicated	Quantitative	Primary	Survey	No	Convenience
**26**	Prozesky, 2008	Unspecified	SA	Unspecified	Faculty	16	55	Qualitative	Primary	Interviews	No	Purposive
**27**	Prozesky, 2006	Multi-HEI national	SA	Unspecified	Faculty	6763	Not indicated	Quantitative	Secondary	Interviews	No	Convenience
**28**	Van Staden *et al*., 2001	Single HEI	SA	HEI	Faculty	19	42.8	Quantitative	Primary	Survey	No	Convenience

***Note:** Only the family name of the first author is indicated. The notation “ *et al.*,” indicates where there are two or more authors.* *N = Number of study participants*

The study population was mainly academic staff (25, 89%). Other participants included librarians (2, 7%) and postgraduate students (1, 3.5%). The mostly applied sampling technique was purposive (10 studies, 36%), followed by the convenient sampling technique (7, 25%) and the stratified random sampling technique (6, 21%). The sample sizes in the studies ranged from eight to 6,763. The average ages of the participants were not reported for most of the studies (22, 79%). However, the average age of the respondents in the eight studies that referred to this variable ranged from 40 to 55 years.

 Although the study targeted the period of 20 years, from 1998 to 2018, the studies that fulfilled the inclusion criteria were mainly published in the last ten years of the covered period (25 papers, 89%), from 2008–2018, among which 17 (61%) papers were published within five years (2013–2018). The duration of the studies was not reported in the majority of the publications (21, 75%). However, out of the nine papers that analysed the study duration, five were conducted in less than one year, while the four were conducted in a period of between one and five years.

Most of the studies (24, 86%) did not indicate the source of funding. The four (14%) that mentioned their source of funding reported that funds came from the researchers' respective higher education institutions. It was not possible to determine how many of the studies had received ethical approval since only six of the publications referred to ethical clearance. Among those, only two reported having received ethical permission, and four indicated that ethical approval did not apply.

Most of the studies (22, 79%) used quantitative methods, six (6, 21%) used qualitative or mixed methods with a dominant qualitative approach. The majority of the studies used primary data (22, 79%), while others utilized secondary (4, 14%) or both primary and secondary data (2, 7%). The data collection methods included surveys (21, 75%), interviews (4, 14%), document analysis (2, 7%), and Focused Group Discussions (FGDs) (1, 3%). Only two of the studies had interventions.

### Operationalization of the concept "research productivity"

In many of the included journal articles, the terms ‘ *research productivity’*, ‘ *research outputs’,* and ‘ *research products’* were used interchangeably. Table 2 shows the different ways in which the concept of research productivity was operationalized in the selected papers.

**Table 2.** Operationalization of research productivity.

Operationalization	Study ID	Number, % of all articles
Journal article publications	[1- 23; 25- 28]	(27, 96%)
Conference presentations	[1- 6; 8- 17; 19- 28]	(26, 93%)
Textbooks	[1; 3; 5-6; 8-19; 21-22; 27]	(19, 68%)
Media presentations	[1, 2, 3, 4, 8, 10, 13, 19, 21]	(9, 32%)
Research grants attracted	[4, 10, 15, 17, 20, 23-24]	(7, 25%)
Technical report	[3, 8, 10, 15, 18, 27]	(6, 21%)
Patent/ Trademark or Innovation	[3, 15, 18, 20]	(4, 14%)
Policy brief	[8, 18]	(2, 7%)
Supervision of Ph.D. students	[3, 15]	(2, 7%)
Blogs	[21]	(1, 4%)

The majority of the studies (27, 96%) operationalized research productivity as journal article publications, followed by conference presentations (26, 93%), textbooks (19, 68%), and media presentations (9, 32%). Other research products included research grant attractions, technical reports, patents/ trademarks or innovations, policy briefs, supervision of postgraduate students, and blogs. In some of the articles, research productivity is operationalized in multiple ways, for instance, a journal article, conference presentation and textbooks.

### Factors associated with research productivity in higher education institutions in Africa

Table 3 presents the individual-related factors of research productivity, which have further been grouped into three subthemes (i.e., sociodemographic, psychological, individual competencies) with several factors under each. Also, the Table shows the number and percentage of the overall studies that reported significant associations between the factors and research productivity or intensely identified the concept as related to research productivity, and a quotation for illustration.

The most frequently reported significant individual-related factors associated with research productivity were motivations and academic qualifications, both of which were published in 32% of the studies ( Table 3). They were closely followed by gender (29%) and research self-efficacy (21%). Other factors included academic rank and tenure (18%); age, academic discipline and attitudes to research (all reported by 14% of the studies); and the individual's research culture and experience (both published in 11% of the articles).

**Table 3.** Individual-related factors significantly associated with research productivity and sample quotes.

Factors	Study IDs	Number and % of papers	Significant quote
**Sociodemographic**			
**Gender**	[5, 9, 13, 16, 22, 25-27]	(8, 29%)	"[female] have shorter publication career spans and interrupt their research and publication momentum because of family-related demands on their time and energy" ( Prozesky, 2008).
**Age**	[9, 16, 22, 26]	(4, 14%)	“…age, designation, and years of experience have a significant positive relationship with research productivity…” ( Anyaogu & Iyabo, 2014).
**Academic Discipline**	[6, 9, 20, 22]	(4, 14%)	“Scientists in the field of chemistry, biochemistry, pharmacy, and those in the field of plant science, animal science, microbiology were found to be more productive than those in the field of physics, mathematics, and electronics” ( Obembe, 2012).
Psychological			
**Motivation**	[4, 6, 9, 11, 12, 15, 23, 27-28]	(9, 32%)	“Being motivated about research was the most commonly reported enabler of research productivity, across all disciplines and career stages” ( Snowball & Shackleton, 2018).
**Research self-efficacy**	[1, 5, 11, 15, 20, 24]	(6, 21%)	“Years as a researcher and research self-efficacy were found to positively predict the research outputs of academics in this context” ( Callaghan, 2015b).
**Locus of control/** **proper time** **management**	[3, 24, 27-28]	(5, 71%)	Persistence [and proper time management] was a trait that was strongly associated with successful publication and was offered as advice to new staff members in both carrying out research and getting it published ( Van Staden *et al*., 2001).
**Attitude/ perception** **of research**	[2, 15, 23, 27]	(4, 14%)	"…early career academics found that [coaching programmes] help them to change their perceptions of their responsibility to themselves in the crucial area of writing for publication" ( Geber, 2009b).
**Individual Culture of ** **research**	[2, 27-28]	(3, 11%)	"…attitude of researchers towards research activities, the contribution of cultures towards research activities, allocated time for research productivity in the university, the cooperation of the research teams, sustainability support and coordination of faculty development initiatives and provision of research information and authorization for external research are the major cultural constraints to research productivity…"( Okendo, 2018).
**Individual competencies**			
**Academic** **Qualification**	[1, 2, 6, 9, 15- 16, 21-22, 25]	(9, 32%)	Findings indicated that the staff qualifications positively influenced research output the most ( Chepkorir, 2018).
**Academic ranks and** **tenure**	[6, 18, 20, 22, 25]	(5, 18%)	“[… librarians…] their promotion and tenure are tied to publishing and research like their teaching counterparts ( Okonedo & Sarah, 2015)
**Experience as a** ** researcher**	[14, 16, 25]	(3, 11%)	“… it is not the influence of total work experience but the influence of experience as a researcher that is primarily associated with higher levels of research productivity in the form of journal and conference outputs” ( Callaghan, 2015a).

### Institutional-related factors associated with research productivity

Table 4 summarizes the data of the institutional-related factors reported as having a significant association with research productivity. The availability of research funds was the most reported institutional-related factor associated with research productivity (43% of the papers). This was followed by networking and collaborations (36%); institutional support to research and conducive policies (32%); research environment and research time (both reported in 29% of the studies); and research mentorship/coaching and internet connectivity (both published in 21% of the papers). Other institutional-related factors included working with graduate students, teaching workload, training and financial incentives. The least reported institutional factors were the availability of office space; institutional ownership; and the institutional administrative structures, each of which was published in only one study.

**Table 4.** Institutional-related factors associated with research productivity.

Factors	Study IDs	Number and % of the papers	Significant quote
**Research capacity and partnerships**			
**Networking/ Collaborations**	[2-3, 6, 8, 10, 15, 20-21, 24, 28]	(10, 36%)	“The study implied that limited participation by scholars in collaborative networks hinders the creation of new knowledge and lowers scholars’ research productivity” ( Moseti & Mutula, 2016).
**Institutional support for research ** **and conducive policies**	[2, 4, 6, 9-10, 15, 17, 23-24]	(9, 32%)	"…factors such as the level of the university, level of supervision, recruitment and selection policies, disparities among faculties, training, department support; put together as institutional factors, play a greater role in enhancing research productivity in Kenya’s Public Universities” ( Muia & Oringo, 2016)
**Research environment**	[4, 6, 9, 11, 15, 21, 23, 24]	(8, 29%)	“…lack of recognition such as promotion, absence of institutional research journal, poor access to information sources such as internet connectivity, insufficient research facilities, lack of financial incentives, lack of institutional/department support on publication, high publication charges inquired by journals, and poor research and publication atmosphere were agreed upon by about 75% of the respondents” ( Feyera *et al*., 2017).
**Research time**	[2-4, 9, 11, 15, 21, 25]	(8, 29%)	“While most of them agreed that the time accorded to the research function was sufficient since they only had to teach for a minimum of ten hours per week, they had reservations about the quality of the institutional infrastructure” ( Musiige & Maassen, 2015).
**Research mentorship/ coaching**	[8, 15, 17, 23-24, 28]	(6, 21%)	“Mentorship and guidance of doctoral students is another organizational component attached to research at MAK [Makerere University].” ( Musiige & Maassen, 2015)
**Working with graduate students**	[3, 6, 9, 15]	(4, 14%)	The role of doctoral students in supporting academics to publish and execute different projects cannot be overstated in research universities” ( Musiige & Maassen, 2015).
**Research Training/ Short Courses**	[2, 6, 9]	(3, 11%)	"Research culture development requires a significant allocation of resources for training and development." ( Okendo, 2018)
**Research funding**			
**Research grants**	[2-4, 6, 9, 11, 15, 17-18, 20-21, 28]	(12, 43%)	The nature of research projects was mainly influenced by donor funding, which usually came with a financial reward for the academics” ( Musiige & Maassen, 2015).
**Financial incentives to encourage** ** research**	[3, 6, 9]	(3, 11%)	“The most cited barriers in order of higher frequency include lack of recognition such as promotion, absence of institutional research journal, poor access to information sources such as internet connectivity, insufficient research facilities, lack of financial incentives, lack of institutional/department support on publication, high publication charges inquired by journals, and poor research and publication atmosphere […]” ( Feyera *et al*., 2017)
**Bureaucracy in funds ** **management and procurement**	[9, 15]	(2, 7%)	“Other concepts and issues that were stated to have an impact on research productivity included […] sophisticated procurement procedures” ( Muia & Oringo, 2016).
**Infrastructural research enabling support**			
**Internet connectivity**	[6, 10, 13, 15, 18, 19]	(6, 21%)	The barriers to research productivity by teaching faculty members in the universities include low Internet bandwidth (M=3.793; SD=1.162) ( Okiki, 2013b).
**Teaching workload (heavy)**	[2, 9, 17, 25]	(4, 14%)	Another aspect that affects research output is workload. Academic staff with a heavy workload of either teaching or administration find it difficult to create time to undertake research ( Nyaribo, 2014).
**Office space**	[4]	(1, 4%)	“The study identified challenges encountered by university faculty members while undertaking research and scholarly publishing. This was evidenced by ineffective documentation of publications, inadequate or no funding at all, poor research infrastructure, inadequate working space, and inadequate time for undertaking research” ( Weng’ua *et al*., 2017).
**Institutional Ownership**	[16]	(1, 4%)	"…ownership of the university significantly correlated positively with research productivity…" ( Anyaogu & Iyabo, 2014)
**Institutional administrative** ** structure**	[15]	(1, 4%)	“…the failure to coordinate the support activities of various central administrative offices resulted in an environment that was not conducive to research work” ( Musiige & Maassen, 2015)

### Research motivations in higher education institutions in Africa

Table 5 shows the papers that focused on the motives for conducting research. The motives for conducting research was greatly attributed to the availability of research funding (43%); followed by the need for a salary increment (25%), and the need to gain recognition and reputation within the academic career (21%). Job satisfaction was the least reported motivation for research, with only two (2, 7%) studies having considered it as a motivating factor for conducting research.

**Table 5.** The motivation for conducting research.

Reasons	Study IDs	Number and % of the papers
Research funding	[2-4, 6, 9, 11, 15, 17, 18, 20-21, 28]	(12, 43%)
Academic promotion and earn extra income or increased salary	[2- 4, 6, 9, 11, 15, 21]	(7, 25%)
Recognition and reputation (including tenure and promotions)	[4, 6, 9, 11, 12, 15, 28]	(6, 21%)
Job satisfaction	[5, 9, 11-12]	(4, 14%)

### Results of the assessment of the risk of bias

**Figure 2.**
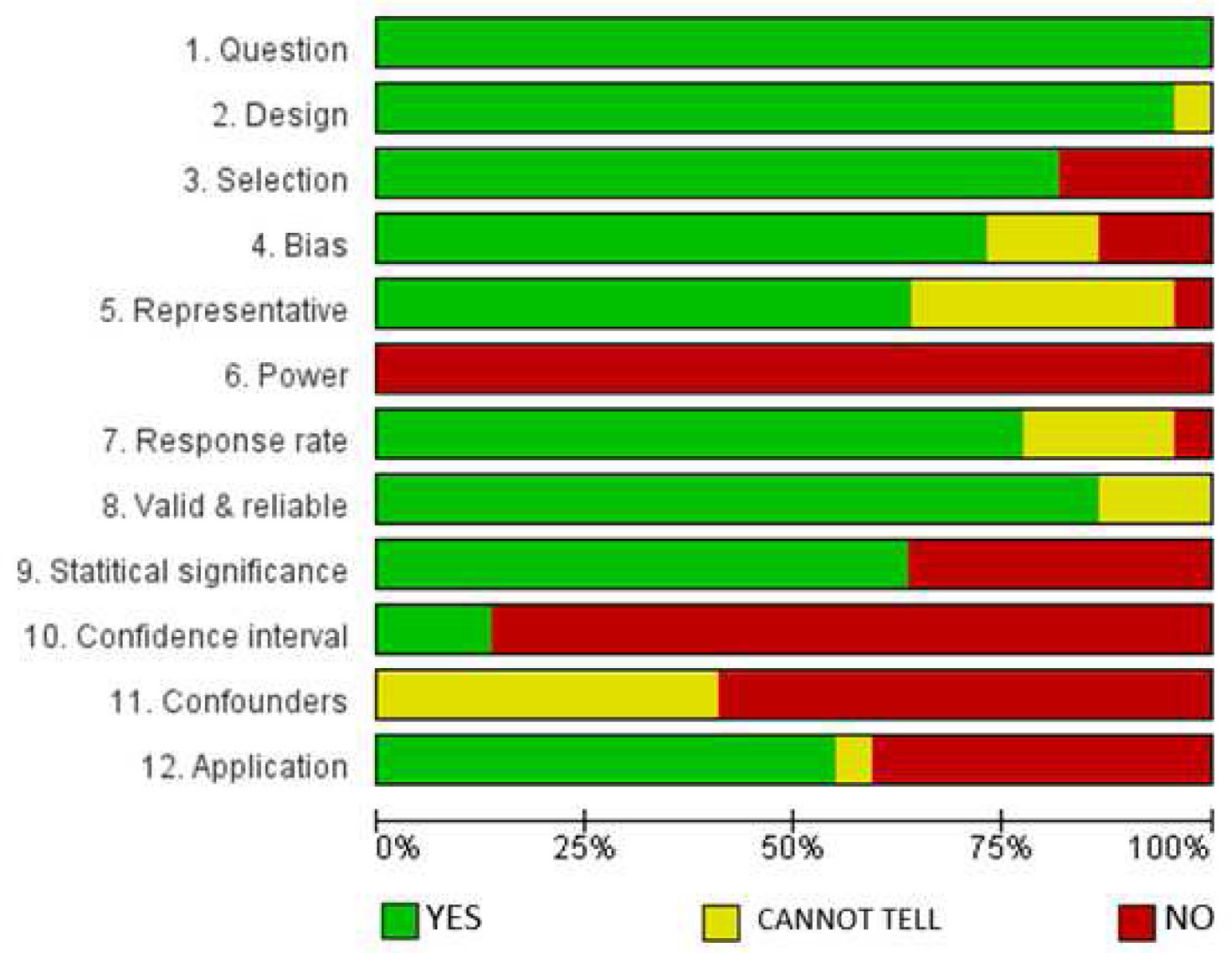
Assessment of methodological quality of quantitative and mixed methods studies.

Figure 2 presents the results of the methodological quality assessment of the quantitative (22) studies included in the analysis, presented using the Cochrane Review Manager tool (RevMan 5.3) ( Uwizeye *et al.*, 2021).

The evaluation ( Figure 2) indicates that all the included articles addressed a clear research question(s). Also, 90% of the papers employed appropriate designs; 80% clearly described selection processes; 75% had low selection bias risk, and 78% had satisfactory response rates, while 85% had high validity and reliability potentials. However, none of the studies based their sample sizes on pre-study conditions of statistical power and most of the studies (over 75%) did not provide confidence intervals or identified confounding factors. The latter results were expected, based on the fact that most of the studies were descriptive and mostly used the purposive sampling technique to identify respondents.

**Figure 3.**
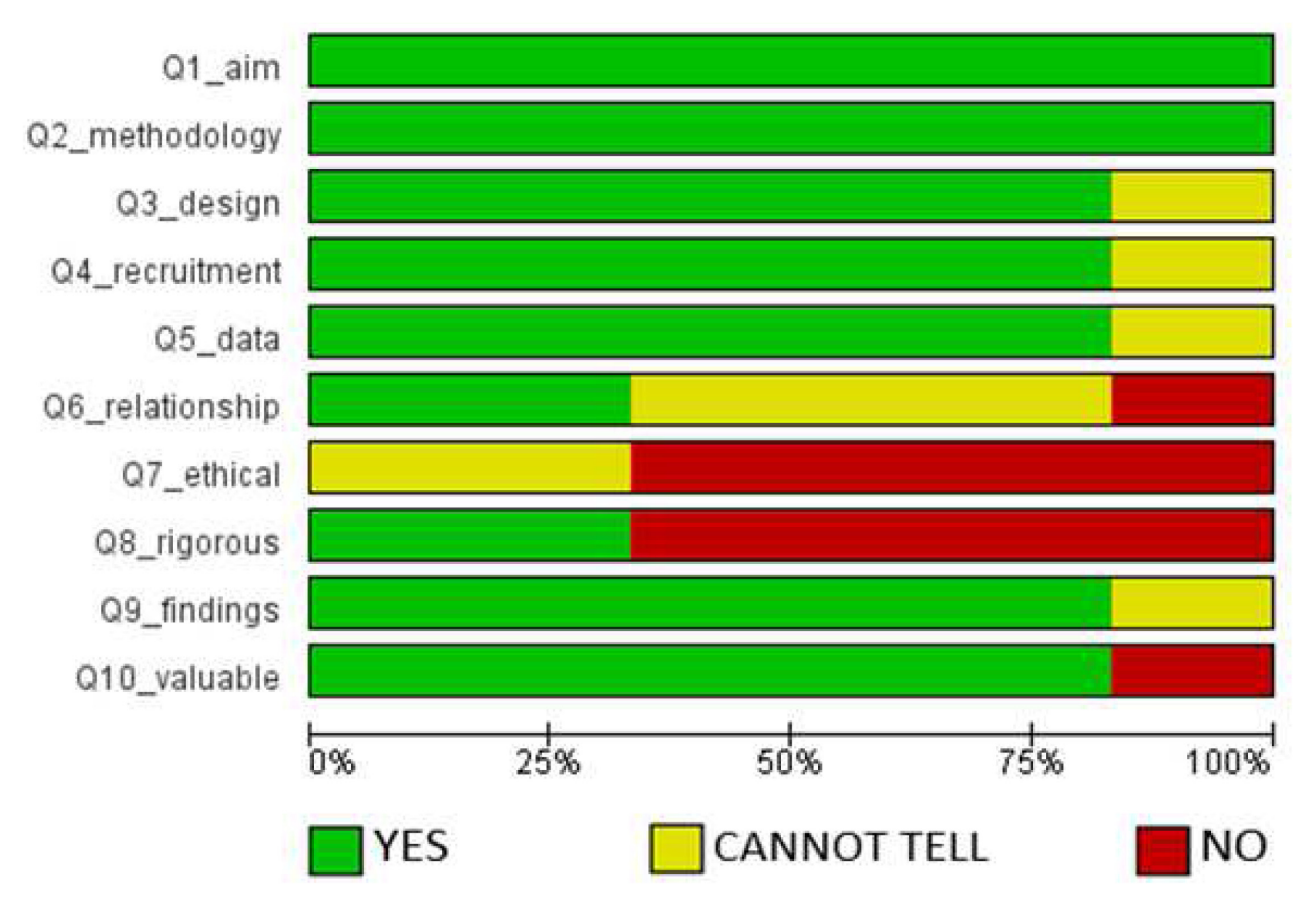
Assessment of methodological quality of qualitative studies.

Similarly, the assessment of the methodological quality of the six qualitative studies is provided in Figure 3 ( Uwizeye *et al.*, 2021).

According to the evaluation presented in Figure 3, all the papers showed a clear statement of the aims of the research, and utilized appropriate qualitative methodology. Over 75% of the publications used proper research design to address the objectives of the study; appropriate recruitment strategies for the informants; and adequate data collection techniques. They also presented clear statements of findings and had the potential to contribute to existing knowledge. On the other hand, less than 50% of the studies clearly described the relationship between the researcher and participants, and very few of them sought ethical clearance before conducting research.

## Discussion

The purpose of this study was to determine the factors associated with research productivity in HEIs in Africa and to identify the motives for conducting research. The study revealed that interest in factors associated with research productivity in HEIs in Africa was progressively increasing with most of the studies having been conducted over the last five years. The review also showed that the highest concentration of the research around the topic was in three countries, South Africa, Nigeria and Kenya, which constitutes less than 5.5% of the number of countries within the African region. Generally, this follows the trend in the overall research productivity in Africa ( Saric *et al.*, 2018). The low percentage in the number of countries that have conducted studies on the factors contributing to research productivity in HEIs raises apprehension on the importance attached to research productivity in the majority of the countries within the broader African region ( Atuahene, 2011; O’Connell *et al.*, 2014; Whitworth *et al.*, 2008), and the gravity is given to the role that research plays in the development process of the continent ( Karimi, 2015). Earlier studies made a similar conclusion on the continental imbalance in research collaborations and publication. African Anglophone countries published more than other parts of the continent ( Adams *et al.*, 2014), and were more likely to engage in research collaborations ( Kabiru *et al.*, 2014).

The results revealed that academic qualifications, motivations, gender and research self-efficacy, were the most reported individual-related factors related to research productivity in African HEIs, and these factors were identified in a similar review ( Mantikayan & Abdulgani, 2018). Elsewhere, researchers argued that raising lecturers self-esteem contributed to increased research productivity ( van Lankveld *et al.*, 2017). Further, retreats provided staff with protected time and space, and opportunities to develop writing competences. Reviews of the interventions that targeted to increase research productivity indicated a positive effect of writing courses, writing support groups and writing coaches ( McGrail *et al.*, 2006) which were also part of the institutional factors. Similarly, Kornhaber *et al.* (2016) discussed that institutions that organized writing retreats and follow up mechanisms increased publication outputs.

Furthermore, research funding and infrastructural research enabling support were reported in many studies as the motivation for research in higher education institutions in Africa. Studies indicated that remuneration and other monetary rewards served as an incentive for scholars to engage in research ( Nguyen, 2015). The study also identified the need for salary increments, availability of scholarly resources, the need for recognition as well as the need to safeguard one’s reputation to be additional motivations for research, beyond research funding, all of which relate to institutional factors. This concurs with the perspective of Musiige & Maassen (2015) who argues that the effectiveness of motivations in research productively depends on the institutional culture on research, which relates to the institutional-related factors of this study, an opinion also held by Feyera *et al.* (2017).

We recognize that this study considered studies of different methodological approaches of qualitative and quantitative studies as observed from the results of the assessment of the risk of bias ( Figure 2 and Figure 3). These factors could potentially have a bearing on the data we harvested, and the conclusions we have made to some extent. However, we remain convinced that the meaning of the findings, and the rationale of the study of informing efforts to increasing research productivity in HEIs in Africa remain significant.

## Conclusion and recommendation

The study concludes that studies that investigated the dearth of research productivity in higher education institutions in Africa remain low and imbalanced. Based on the available studies, institutional factors are more attributed to research productivity than individual-related factors. More specifically, factors such as enhanced faculty research networks and collaborations, and research supporting policies offered protected research time to faculty members and created a conducive research environment that motivated researchers to increase research productivity.

The study recommends that the leadership of higher education institutions in Africa invests in funding research for researchers to contribute to the continental development agenda. Also, institutional support to research, including the provision of research enabling environments and policies; provision of research output incentives; strengthening of researchers’ capabilities through relevant training courses, and provision of opportunities for mentorship and coaching should be strengthened. Besides, higher education institutions in Africa should develop secure institutional research networks and collaborations.

## Data availability

### Underlying data

Open Science Framework: Factors associated with research productivity in higher education institutions in Africa: a systematic review. https://doi.org/10.17605/OSF.IO/P3GVX ( Uwizeye *et al.*, 2021).

This project contains the following extended data:

- Raw data_ ROBA_ for qualitative papers
- Raw data_ ROBA_ for quantitative papers

### Extended data

Open Science Framework: Factors associated with research productivity in higher education institutions in Africa: a systematic review. https://doi.org/10.17605/OSF.IO/P3GVX ( Uwizeye *et al.*, 2021).

This project contains the following extended data:

- Characteristics of the Analysed studies
- Protocol for the review
- Results of the assessment of the risk of bias
- Search strategy 2_ an example with Google Scholar
- Search Strategy__ Example (example outcome of search with EBSCOHost)
- Supplementary Table 2__ Included Studies

### Reporting guidelines

PRISMA checklist for "Factors associated with research productivity in higher education institutions in Africa: a systematic review" available: https://doi.org/10.17605/OSF.IO/P3GVX ( Uwizeye *et al.*, 2021). 

Data are available under the terms of the Creative Commons Attribution (CC0 1.0 Universal). 
