# Supplementary material for: Factors associated with research productivity in higher education institutions in Africa: a systematic review
Source: AAS Open Res. 2022 Jan 28;4:26. Originally published 2021 May 26. [Version 2] doi: 10.12688/aasopenres.13211.2 (PMC8311799; doi:10.12688/aasopenres.13211.2)
Supplement: Peer review history

# Peer review: Reviewer response for version 1

**DOI:** 10.21956/aasopenres.14328.r28734

**Published:** 2021-07-21

**Authors:** Rose Okoyo Opiyo

**Affiliations:** 1 University of Nairobi, Nairobi, Kenya

This review has been conducted with rigor and is well written. However, I have **minor comments** which the author can address and the paper can be accepted for indexing.

**Background:** Research is a core mandate of HEIs (Jowi *et al.*, 2013 ^1^). Please articulate this in the background. Yet, key output of post-graduate students’ research has been thesis/dissertation, and less focus on publications as shown in the review. 

**Methods:**

- On the PRISMA diagram at Screening stage, add the word **“abstracts”** so that you have “Records titles and abstracts screened (n=838) and “Records titles and abstracts excluded (n=766).
- Column 2, 2 ^nd^ paragraph, Line 1: Edit to read – “The papers included in this review were both quantitative and qualitative.” This paragraph should also be part of the previous paragraph after the Galdas *et al.*, 2015 citation.
- I am just wondering whether we can replace the phrase “the included papers…” with “the papers included in the review...” where applicable.
- Although the review protocol does not seem to have been registered, it is available under extended data.

**Results: **

- 3 ^rd^ line  - delete “the search of” – it is redundant.
- 4 ^th^ Line – after 838 – add “papers”.
- Table 1: Define “Location” under Table legend to mean “single institution or multiple institution–based”.
- Page 5, Column 2, 1 ^st^ sentence: An interesting finding that research output from postgraduate students is very minimal. Or does it mean that faculties were the corresponding authors? I hope this is picked up in the discussion section.
- Page 5 Column 2, 2nd sentence: “The mostly applied sampling…” needs rephrasing.
- Is the “age of researcher/authors” a common feature in research publications? If not, then I don’t think this is necessary here.

**Discussion: **

- The review found that most studies were published between 2008 and 2018 and not before, yet, the review period was 1998 to 2018. The period when research became a core mandate for HEIs in Africa might have contributed to the observed findings here. Any comment on this under discussion?
- This review also found that most studies lacked ethical clearance and statistical power. Furthermore, only about 30% of the reviewed studies were found to be rigorous enough to allow meaningful conclusions to be drawn. The finding on poor quality of manuscripts might have contributed to their rejection at journals submission stage, thus contributing to low productivity **. The authors need to discuss this.**
- Operationalization of research productivity concept: Is it possible to include a paragraph or sentence on implication of operationalization of the concept for national/global development agenda?
- In addition to motivating factors, is it possible to highlight key barriers too in results and discussion.

Are the rationale for, and objectives of, the Systematic Review clearly stated?

Yes

Is the statistical analysis and its interpretation appropriate?

Yes

Are sufficient details of the methods and analysis provided to allow replication by others?

Yes

Are the conclusions drawn adequately supported by the results presented in the review?

Yes

Reviewer Expertise:

Public Health and Nutrition research

I confirm that I have read this submission and believe that I have an appropriate level of expertise to confirm that it is of an acceptable scientific standard.

## References

[1] : Governance of higher education, research and innovation in Ghana, Kenya and Uganda (Part of a report: Programme on Innovation, Higher Education and Research for Development, IHERD). *Paris, France: OECD* .

# Response

**Published:** 2021-12-27

**Authors:** Dieudonne Uwizeye

**Affiliations:** University of Rwanda, Rwanda

**Comment# 1:**

 This review has been conducted with rigor and is well written. However, I have **minor comments** which the author can address and the paper can be accepted for indexing.

**Response:**

 Thank you for the comments. We produced the revised version of the manuscript in consideration of the comments.

**Comment# 2:**

**Background: **Research is a core mandate of HEIs (Jowi *et al.*, 2013 ^1^). ****Please articulate this ****in the background. ****Yet, the key output of post-graduate students’ research has been thesis/dissertation, and less focus on publications as shown in the review. 

**Response:**

 Thank you for the comment. We have extended our background to emphasize that the HEIs’ core mandate includes research. Also, we argued how some of the HEIs in Africa do not enforce the research productivity by the postgraduate students. We argued that this practice may compromise the increase of research productivity in HEIs in Africa.  

**Comment# 3:**

**Methods:**

- On the PRISMA diagram at Screening stage, add the word **“abstracts”** so that you have “Records titles and abstracts screened (n=838) and “Records titles and abstracts excluded (n=766). 
- Column 2, 2 ^nd^ paragraph, Line 1: Edit to read – “The papers included in this review were both quantitative and qualitative.” This paragraph should also be part of the previous paragraph after the Galdas *et al.*, 2015 citation. 
- I am just wondering whether we can replace the phrase “the included papers…” with “the papers included in the review...” where applicable. 
- Although the review protocol does not seem to have been registered, it is available under extended data.

**Response:**

 The editorial issues raised above were meaningful. They were addressed in the revised version of the paper. 

**Comment# 4:**

**Results:**

- 3 ^rd^ line  - delete “the search of” – it is redundant. 
- 4 ^th^ Line – after 838 – add “papers”. 
- Table 1: Define “Location” under Table legend to mean “single institution or multiple institution–based”. 
- Page 5 Column 2, 2nd sentence: “The mostly applied sampling…” needs rephrasing.

**Response:**

 The editorial issues raised above were meaningful. They were addressed in the revised version of the paper. 

**Comment# 5:**

- Page 5, Column 2, 1 ^st^ sentence: An interesting finding that research output from postgraduate students is very minimal. Or does it mean that faculties were the corresponding authors? I hope this is picked up in the discussion section.
-  Is the “age of researcher/authors” a common feature in research publications? If not, then I don’t think this is necessary here.

**Response:**

 Thank you for these two comments.

 Postgraduate students described in the indicated passage were the participants in the studies we reviewed and not the papers by postgraduate students. To put it plainly, this systematic review paper indicated evidence that papers that interviewed postgraduate students about the “factors and motivations” of research productivity in HEIs in Africa were very minimal. This implies that most of the available information about “factors and motivations” of research productivity in HEIs in Africa mainly came from lecturers.

 Similarly, the variable “age” is not the age of the authors that wrote the papers we reviewed. Rather, it is the age of the respondents in the papers we reviewed.

 We thank the reviewer for these two comments because we considered them in the revised version to ensure that the readers are not confused in the future.   

**Comment# 6: Discussion: **The review found that most studies were published between 2008 and 2018 and not before, yet, the review period was 1998 to 2018. The period when research became a core mandate for HEIs in Africa might have contributed to the observed findings here. Any comment on this under discussion?

**Response: **Thank you for the comment. We made it clear in the revised version that research has always been one of the core mandates of HEIs in Africa. The 20 years period was selected to capture the changes that happened over the years. One of the changes we observed and discussed in the paper was that the interest to understand the factors and motives of research productivity within HEIs in Africa is relatively recent. This is an important finding and it informs researchers to do more research on this to inform efforts to address the challenges addressed. 

**Comment# 7: **This review also found that most studies lacked ethical clearance and statistical power. Furthermore, only about 30% of the reviewed studies were found to be rigorous enough to allow meaningful conclusions to be drawn. The finding on the poor quality of manuscripts might have contributed to their rejection at the journals submission stage, thus contributing to low productivity. The authors need to discuss this.

**Response: **Thank you for the comment. ****We argued that most of the studies were descriptive, and used purposive sampling as they were targeting a specific population for data collection. Understandably, the studies were not expected to have statistically selected their samples and, therefore, the presentation of the statistical power was not expected.  This comment applies to other statistical measures like the confidence intervals and the consideration of confounders as well.

 Further, we appreciate the comment on ethical clearance. We extended the argument on this to indicate that we were not surprised that most of the studies did not comment on ethical clearance. Usually, seeking ethical clearance is not a compulsory practice in education research. 

     

**Comment# 8: **Operationalization of research productivity concept: Is it possible to include a paragraph or sentence on the implication of operationalization of the concept for national/global development agenda? In addition to motivating factors, is it possible to highlight ****key barriers too in results and discussion.

**Response:**

 Thank you for the comment.

 We collected data about how the concept of “research productivity” was operationalized in the papers we reviewed to inform readers of the meaning of the concept in the paper, and its driving factors in HEIs in Africa. We believe that we have achieved this purpose. Linking the conceptualization of the concept with its implication on the national/global development agenda was not aimed at its own.

 On the comment about highlighting the key barriers to research productivity, this paper opted the line of reviewing and discussing the factors associated with research productivity, and the motives of doing research in HEIs in Africa. In doing so, the barriers to research productivities are inferred. We believe that readers will find it easy to understand the barriers to research productivity by reading the driving factors and motives to it.

**Comment# 9:**

- Are the rationale for, and objectives of, the Systematic Review clearly stated?

**Yes**

- Are sufficient details of the methods and analysis provided to allow replication by others?

**Yes**

- Is the statistical analysis and its interpretation appropriate?

**Yes**

- Are the conclusions drawn adequately supported by the results presented in the review?

**Yes**

**Response:**

 We really appreciate the comments from the reviews. In consideration of the comments, we revised and published the revised version of the paper.

# Peer review: Reviewer response for version 1

**DOI:** 10.21956/aasopenres.14328.r28674

**Published:** 2021-07-19

**Authors:** Millicent L. Liani

**Affiliations:** 1 Liverpool School of Tropical Medicine, Liverpool, UK; 2 University of Liverpool, Liverpool, UK

Many thanks for the opportunity to review this article. It was a pleasurable experience reading it! Great efforts by the authors to describe a research area that has been largely anecdotally explored. The paper aims to provide an understanding of factors associated with research productivity in higher education institutions in Africa. It is well written in English and discussed in detail.

 I have very minor comments and suggestions:

 1) In the introduction section, the authors note that the purpose of the study is two-fold – To: determine factors associated with research productivity in HEIs in Africa, and identify what motivates researchers working in HEIs in Africa to do research. However, in the methods section, there is no mention of the latter rationale (motivational factors) within the inclusion and exclusion criteria. They could also add this aim to the scope of the research papers.

 2) The last sentence in the introduction section can be moved to the methods section at the first place. Table 1: ‘study characteristics’ should be edited to address typographic errors i.e. interview, FDG, and could be treated as an appendix/supplementary file. Table 3 and 4 requires further formatting i.e. the key factors such as sociodemographic, psychological and individual competencies, research capacity and partnerships, research funding etc should be in bold, while the sub-factors shouldn’t be written in bold font.

 3) There is mention of French language as inclusion criteria for selection of review papers, yet the authors do not refer to this in the results and discussion section. Notably, they highlighted that African Anglophone countries publish more than other parts of the continent and are likely to engage in research. Some detailed insights on challenges faced by African Francophone countries in relation to research productivity could help strengthen the paper. The articles below focus on why English as a standard language for scientific research communication presents challenges for many non-Anglophone speaking scientists, but can give you some pointers: 

- Hyland (2016 ^1^). 
- Martín *et al.* (2014 ^2^).

4) In the discussion section, the authors capture some very important perspectives around individual level factors associated with research productivity which includes academic qualifications, motivation and gender. I think some further nuanced reflections on how these factors relates to research productivity would greatly strengthen the claims being made in the paper. In addition, they should consistently use the abbreviation HEIs in the discussion and conclusion sections as earlier utilised in the previous sections.

 I look forward to seeing this work indexed!

Are the rationale for, and objectives of, the Systematic Review clearly stated?

Yes

Is the statistical analysis and its interpretation appropriate?

Yes

Are sufficient details of the methods and analysis provided to allow replication by others?

Yes

Are the conclusions drawn adequately supported by the results presented in the review?

Yes

Reviewer Expertise:

Gender research; Scientific research capacity strengthening in higher education and research institutions in Africa

I confirm that I have read this submission and believe that I have an appropriate level of expertise to confirm that it is of an acceptable scientific standard.

## References

[1] : Academic publishing and the myth of linguistic injustice. *Journal of Second Language Writing* .2016;31: 10.1016/j.jslw.2016.01.005 58-69 10.1016/j.jslw.2016.01.005 PMC550276128701854

[2] : Publishing research in English-language journals: Attitudes, strategies and difficulties of multilingual scholars of medicine. *Journal of English for Academic Purposes* .2014;16: 10.1016/j.jeap.2014.08.001 57-67 10.1016/j.jeap.2014.08.001

# Response

**Published:** 2021-12-27

**Authors:** Dieudonne Uwizeye

**Affiliations:** University of Rwanda, Rwanda

**Comment# 1:**

 Many thanks for the opportunity to review this article. It was a pleasurable experience reading it! Great efforts by the authors to describe a research area that has been largely anecdotally explored. The paper aims to provide an understanding of factors associated with research productivity in higher education institutions in Africa. It is well written in English and discussed in detail.

 I have very minor comments and suggestions:

**Response:**

 Thank you. We appreciated the comments; they helped us to revise the manuscript.

**Comment# 2:**

 1) In the introduction section, the authors note that the purpose of the study is two-fold – To: determine factors associated with research productivity in HEIs in Africa, and identify what motivates researchers working in HEIs in Africa to do research. However, in the methods section, there is no mention of the latter rationale (motivational factors) within the inclusion and exclusion criteria. They could also add this aim to the scope of the research papers.

**Response:**

 Thank you for the comment. We have added a sentence to extend the purpose of the study.

**Comment# 3:**

 2) The last sentence in the introduction section can be moved to the methods section at the first place. Table 1: ‘study characteristics’ should be edited to address typographic errors i.e. interview, FDG, and could be treated as an appendix/supplementary file. Table 3 and 4 requires further formatting i.e. the key factors such as sociodemographic, psychological and individual competencies, research capacity and partnerships, research funding etc should be in bold, while the sub-factors shouldn’t be written in bold font.

**Response:**

 Thank you for the observations. All of them are valid, and we have revised the paper accordingly. The corrections are published in the revised version of the paper.

**Comment# 4:**

 3) There is mention of French language as inclusion criteria for the selection of review papers, yet the authors do not refer to this in the results and discussion section. Notably, they highlighted that African Anglophone countries publish more than other parts of the continent and are likely to engage in research. Some detailed insights on challenges faced by African Francophone countries in relation to research productivity could help strengthen the paper. The articles below focus on why English as a standard language for scientific research communication presents challenges for many non-Anglophone speaking scientists but can give you some pointers: 

- Hyland (2016 ^1^ ). 
- Martín *et al.* (2014 ^2^ ).

**Response:**

 Thank you for the comment. We considered this comment and added additional information in the discussion. The provided references are very much related to the additional discussion and we have cited them and enriched our list of references.

**Comment# 5:**

 4) In the discussion section, the authors capture some very important perspectives around individual-level factors associated with research productivity which includes academic qualifications, motivation and gender. I think some further nuanced reflections on how these factors relate to research productivity would greatly strengthen the claims being made in the paper. In addition, they should consistently use the abbreviation HEIs in the discussion and conclusion sections as earlier utilised in the previous sections.

**Response:**

 We added an extension of our discussion of the academic qualifications, motivation and gender to indicate how these factors are linked with academic research productivity.

 Also, we have consistently used the abbreviation HEIs where it is appropriate throughout the paper.

**Comment# 6: **

- **Are the rationale for, and objectives of, the Systematic Review clearly stated?**

Yes

- **Are sufficient details of the methods and analysis provided to allow replication by others?**

Yes

- **Is the statistical analysis and its interpretation appropriate?**

Yes

- **Are the conclusions drawn adequately supported by the results presented in the review?**

Yes

**Response**

 Thank you very much for reviewing our manuscript. It gave us the opportunity to produce the revised version.

# Peer review: Reviewer response for version 1

**DOI:** 10.21956/aasopenres.14328.r28631

**Published:** 2021-07-15

**Authors:** Ojo Melvin Agunbiade

**Affiliations:** 1 Department of Sociology and Anthropology, Obafemi Awolowo University, Ife, Nigeria

**General:** The review addresses a relevant gap in evidence synthesis on research productivity within African higher education institutions. This review marshals critical factors at both individual and institutional levels and how these factors intersect to influence research productivity within the higher education context. There is a good sense of rigor in the review process and readers could see how the review was conducted following the established standard. However, a few editorial issues require the attention of the authors.

**Editorial:**

- There are editorial issues in some sections of the paper.

**Abstract:**

- Check the sentence under the inclusion criteria II, and the concluding sentence under the conclusion.

**Introduction:**

- First paragraph needs some copy-editing. There is also a need to look for a more appropriate term to replace: "university faculty members" in line 3 from the bottom of the paragraph.
- Second paragraph: Reconsider the third sentence in this paragraph (e.g. Africa populations are huge, yet only few researchers are active and making impact. Evidence on numerical strength shows that only Morocco has more than 1000 researchers per one million inhabitants.)
- Third paragraph: consider replacing the word "determine" in the sentence: "Therefore, there is an urgent need to determine…". (Consider: ...need to synthesize evidence on the factors...).
- Also consider replacing the word study in the sentence that begins with: "The purpose of this study..." with a word like systematic review.

**Methods:**

- Analysis approach - the second paragraph should start with an inter connector to allow a flow between this paragraph and the previous one.

Are the rationale for, and objectives of, the Systematic Review clearly stated?

Yes

Is the statistical analysis and its interpretation appropriate?

Partly

Are sufficient details of the methods and analysis provided to allow replication by others?

Yes

Are the conclusions drawn adequately supported by the results presented in the review?

Yes

Reviewer Expertise:

sociology of health, sexual health in old age, social policy, help-seeking behaviour and mixed methods designs

I confirm that I have read this submission and believe that I have an appropriate level of expertise to confirm that it is of an acceptable scientific standard, however I have significant reservations, as outlined above.

# Response

**Published:** 2021-12-27

**Authors:** Dieudonne Uwizeye

**Affiliations:** University of Rwanda, Rwanda

**Comment# 1**

**General:** The review addresses a relevant gap in evidence synthesis on research productivity within African higher education institutions. This review marshals critical factors at both individual and institutional levels and how these factors intersect to influence research productivity within the higher education context. There is a good sense of rigor in the review process and readers could see how the review was conducted following the established standard. However, a few editorial issues require the attention of the authors.

**Response:**

 Thank you for the review. The indicated editorial issues and others were addressed in version two of the paper.

**Comment#2:**

**Editorial:**

 There are editorial issues in some sections of the paper.

**Abstract:**

 Check the sentence under the inclusion criteria II, and the concluding sentence under the conclusion.

**Introduction:**

*First paragraph needs some copy-editing*. There is also a need to look for a more appropriate term to replace: "university faculty members" in line 3 from the bottom of the paragraph.

*Second paragraph*: Reconsider the third sentence in this paragraph (e.g. Africa populations are huge, yet only few researchers are active and making impact. Evidence on numerical strength shows that only Morocco has more than 1000 researchers per one million inhabitants.) 

*Third paragraph*: **consider replacing the word "determine" in the sentence: "Therefore, there is an urgent need to determine…". (Consider: ...need to synthesize evidence on the factors...). 

 Also consider replacing the word study in the sentence that begins with: "The purpose of this study..." with a word like systematic review.

**Methods:**

 Analysis approach - the second paragraph should start with an inter connector to allow a flow between this paragraph and the previous one.

**Response:**

 All the editorial issues indicated above were considered and addressed in the revised version of the manuscript.

**Comment# 3:**

- Are the rationale for, and objectives of, the Systematic Review clearly stated?

**Yes**

- Are sufficient details of the methods and analysis provided to allow replication by others?

**Yes**

- Is the statistical analysis and its interpretation appropriate?

Partly

- Are the conclusions drawn adequately supported by the results presented in the review?

**Yes**

**Response:**

 Thank you. We appreciate the time and energy you offered to review our manuscript. The comments and observations you made are very valuable, and they helped us to produce the revised version of the paper. 

 For the question of whether the statistical analysis and its interpretation were appropriate, we want to explain that this was not appropriate.
